# Stop stunting: improving child feeding, women's nutrition and household sanitation in South Asia

**DOI:** 10.1111/mcn.12283

**Published:** 2016-05-17

**Authors:** Víctor M. Aguayo, Purnima Menon

**Affiliations:** ^1^ Regional Nutrition Advisor for South Asia, United Nations Children's Fund (UNICEF), Regional Office for South Asia Kathmandu Nepal; ^2^ Senior Research Fellow, International Food Policy Research Institute (IFPRI) New Delhi India

**Keywords:** stunting, child feeding, women's nutrition, household sanitation, South Asia

## Abstract

The latest available data indicate that 38% of South Asia's children aged 0–59 months are stunted. Such high prevalence combined with the region's large child population explain why South Asia bears about 40% of the global burden of stunting. Recent analyses indicate that the poor diets of children in the first years of life, the poor nutrition of women before and during pregnancy and the prevailing poor sanitation practices in households and communities are important drivers of stunting, most likely because of underlying conditions of women's status, food insecurity, poverty, and social inequalities. With this evidence in mind, UNICEF Regional Office for South Asia convened the Regional Conference: *Stop Stunting*: *Improving Child Feeding*, *Women*'*s Nutrition, and Household Sanitation in South Asia* (New Delhi, November 10–12, 2014). The Conference provided a knowledge‐for‐action platform with three objectives: (1) share state‐of‐the‐art research findings on the causes of child stunting and its consequences for child growth and development and the sustainable growth and development of nations; (2) discuss better practices and the cost and benefits of scaling up programmes to improve child feeding, women's nutrition, and household sanitation in South Asia; and (3) identify implications for sectoral and cross‐sectoral policy, programme, advocacy and research to accelerate progress in reducing child stunting in South Asia. This overview paper summarizes the rationale for the focus on improving child feeding, women's nutrition, and household sanitation as priority areas for investment to prevent child stunting in South Asia. It builds on the invited papers presented at or developed as a follow on to the Stop Stunting Conference.

**Key messages**

- More than a third of South Asia's children aged 0–59 months are stunted because of persistent nutritional deprivation. South Asia bears about 40% of the global burden of stunting.
- Three important drivers of child stunting in South Asia are the poor diets of children in the first 2 years of life, the poor nutrition of women before and during pregnancy, and poor sanitation practices and conditions in households and communities.
- Economic growth alone will not improve stunting without commensurate strategic investments in evidence‐based large scale programmes that place their emphasis on closing equity gaps, especially for the most vulnerable children and populations.

## Child stunting in South Asia. Why does it matter?

The linear growth of healthy children from birth to five years of age is remarkably similar the world over (Multicenter Growth Reference Study Group 2006). Yet, the latest global figures indicate that ~25% of children under age five (i.e. 159 million) have stunted growth because of chronic nutrition deprivation (UNICEF, WHO, WBG 2016). Stunting—a height‐for‐age below −2 standard deviations of the median height‐for‐age in the Child Growth Standards of the World Health Organization—happens early in life. There is now broad agreement that most stunting happens during the first thousand days—from conception through the first two years of life—although additional linear growth faltering may still happen after the first two years of life (Leroy *et al*. 2014).

In their paper in this special issue de Onis and Branca, from the World Health Organization, remind us that besides early beginnings, stunting also has far‐reaching consequences (de Onis & Branca 2016). It is estimated that stunting is the cause of about one million child deaths annually. For the children who survive, stunting in infancy and early childhood causes lasting damage, including increased morbidity, poor cognition and educational performance in childhood, short stature in adulthood, increased risk of perinatal and neonatal death for women, lower productivity and reduced earnings in adults and—when accompanied by excessive weight gain later in childhood—increased risk of chronic diseases. Therefore, it is accurate to say that stunting hampers the development of entire societies (Victora *et al*. 2008; Dewey & Begum 2016; Black *et al*. 2013; de Onis & Branca 2016).

The latest available data indicate that 38% of South Asia's children under five years of age are stunted. Levels of child stunting in South Asia are comparable to those in sub‐Saharan Africa (37%) and three times higher than those in East Asia and the Pacific (12%) or Latin America (11%). The high prevalence of stunting and the region's large child population (26% of the world's children under five) means that South Asia, with about 40% of the global burden of stunting, is the epicentre of the global stunting crisis (United Nations Children's Fund, UNICEF 2015, 2015a). Recent analyses indicate that three main drivers of child stunting in South Asia are the poor diets of children in the first years of life, the poor nutrition of women before and during pregnancy and the prevailing poor sanitation practices in households and communities (Smith & Haddad 2016).

With this evidence in mind, UNICEF Regional Office for South Asia convened a regional conference in New Delhi, India under the theme: *Stop Stunting*: *Improving Child Feeding*, *Women*'*s Nutrition and Household Sanitation in South Asia* (November 10–12, 2014). The Conference provided a knowledge‐for‐action platform with three objectives: (1) share state‐of‐the‐art research findings on the causes of child stunting and its consequences for child growth and development and the sustainable growth and development of South Asian nations; (2) discuss better practices and the cost and benefits of scaling up programmes to improve child feeding, women's nutrition and household sanitation in South Asia; and (3) identify implications for sectoral and cross‐sectoral advocacy, policy, programme and research to accelerate progress in reducing child stunting in South Asia.

The Regional Conference was attended by about 200 participants representing national governments and regional organizations as well as bilateral, multilateral and non‐governmental development partners, representatives of research and academic institutions and resource persons from South Asia and globally. This special issue of *Maternal and Child Nutrition* includes the guest presentations made at the Conference and a series of invited papers that were developed in preparation for or as a follow up to the Conference.

## The drivers of child stunting in South Asia

There is emerging consensus that economic growth can have a positive impact on reducing child stunting. However, the relationship between economic growth and child stunting is not always a straightforward one. In this issue, Joe *et al.* investigate why economic growth did not result in a significant reduction of child stunting in India between 1993 and 2006. They conclude that economic growth did not lead to significant increases in public development expenditure, considerable reductions in poverty and/or increased equity, the pathways through which economic growth is expected to reduce stunting in low income and middle income countries (Joe *et al*. 2016).

Applying comparable statistical techniques to Demographic and Health Surveys (DHS) data collected between 1993 and 2013 in Bangladesh, India, Nepal and Pakistan, Headey *et al.* extract a set of explanatory variables that cover a broad range of the hypothesized distal drivers of nutrition change. They find that improvements in household asset accumulation (reduced poverty), combined with improvements in women's education and reductions in open defecation, account for much of the observed nutritional change in height‐for‐age *z*‐scores in children 0–59 months in these four countries. India's relative poorer performance (1993–2006) is driven by smaller changes in these variables (Headey *et al*. 2016).

Lastly, Subramanian *et al.* critique the over‐reliance on macroeconomic growth as a policy instrument to improve nutrition in children and argue that the evidence that an economic growth‐mediated strategy only can reduce child stunting at scale is no longer compelling. They also critique a disproportionate focus on interpreting single‐factorial (often proximal in nature) approaches to reducing stunting. They argue that instead of addressing one risk factor at a time—whether proximal or distal—a support‐led multi‐sectoral approach is needed to stop stunting in South Asia (Subramanian *et al*. 2016).

Focusing on India, Aguayo *et al.* analyse a representative sample of children 0–23 months old to identify the factors most significantly associated with child stunting in the State of Maharashtra. They find that stunting and poor attained linear growth (height‐for‐age *z*‐score) were significantly poorer in children born with low birth weight, children born to mothers whose height was <145 cm, children of mothers without decision making power regarding food, children 6–23 months old who were not fed a minimum number of times per day, children who were not fed eggs, dairy products, fruits and/or vegetables and children from households without access to improved sanitation (Aguayo *et al*. 2016).

Taken together, these papers emphasize that while economic growth is an important imperative for South Asia, particularly to generate government revenues that can be invested in social policies and programmes, it is also equally imperative to make the necessary policy and programme investments that can help to accelerate progress in improving child feeding, women's nutrition and household sanitation. Investments in this region, and in these issues, are critical if the world is to achieve the global target of reducing by 40% the number of children under five who are stunted, from 171 million in 2010 to about 100 million in 2025 (WHO 2015). The papers in this special issue of *Maternal and Child Nutrition* highlight some of the challenges, and more importantly, the successes of attempts to shape these determinants.

## The challenge of improving child feeding

Most stunting happens in the first 1000 days, from conception to age two years, when children's linear growth is most sensitive to nutrition deprivation and environmental stress. During the first 500 days, from conception to about 6  months of age, the child is entirely dependent for its nutrition on the mother, either via the placenta during pregnancy or via breastmilk during the initial 6‐month exclusive breastfeeding period. However, the largest proportion of stunting occurs during the complementary feeding period (6–23 months), the ~500‐day transition time from exclusive breastfeeding in the first 6 months of life, to consuming a wide range of family foods while breastfeeding continues. Adequate complementary feeding is critical to support optimal physical growth and brain development in children. Complementary foods need to be nutrient‐rich and be fed frequently to prevent stunting. The most recent data indicate that fewer than 25% of children 6–23 months old in Afghanistan, Bangladesh, India, Nepal and Pakistan are fed diets that meet the minimum requirements in terms of frequency and diversity (United Nations Children's Fund, UNICEF 2015, 2015a).

Addressing this subject, Dewey reviews in this special issue the various options for improving the diets of pregnant and lactating women and their children in the first two years of life. These options include dietary diversification and increased intake of nutrient‐rich foods for women, improved complementary foods and feeding practices for children and micronutrient supplements, fortified foods and products specifically designed for infants, young children, pregnant women and lactating women. Dewey's review indicates that these interventions, both prenatal and postnatal, can have a positive impact on child growth. However, there is significant heterogeneity in linear growth response to such interventions. Such variation is likely to be related to the potential to benefit (i.e. is the population undernourished?) and the potential to respond to improved nutrition (i.e. are there other factors constraining linear growth?). Hence, the importance of understanding the aetiology of poor linear growth and stunting and the need for integrated approaches that address the potentially multifactorial aetiology of stunting (Dewey 2016).

In their paper, Paintal and Aguayo remind us that optimal infant and young child feeding (IYCF) practices are particularly crucial when children are sick or convalescent as children's nutritional status can deteriorate rapidly if the additional nutrient requirements associated with illness and convalescence are not met and nutrients are diverted from growth and development towards the immune response. Their review of survey and research evidence shows that in South Asia, IYCF practices during common childhood illnesses are far from optimal. Most children continue to be breastfed when they are sick, but few are breastfed more frequently, as recommended. In addition, restriction or withdrawal of complementary foods during illness is frequent because of children's anorexia (perceived or real), poor awareness of caregivers' about the feeding needs of sick children, traditional beliefs/behaviours and/or sub‐optimal counselling and support by health workers (Paintal & Aguayo 2016).

Evidence from the region suggests that large‐scale improvements in IYCF are possible when interventions are designed for and delivered at scale. Sanghvi *et al.* review the experience of the Alive & Thrive programme in Bangladesh, which focused on a population of 8.5 million mothers and their families/communities. After 4 years of implementation, the programme documented rapid and significant improvements in key breastfeeding and complementary feeding practices. Promotion strategies reached a high percent of the priority groups through repeated contacts. Scale‐up was achieved by mainstreaming tools and strategies in the work of government programmes and local NGO implementing partners with an extensive community‐based platform. Improving the performance of frontline workers and volunteers in delivering timely, high‐quality counselling to mothers while reinforcing interpersonal counselling with mass media campaigns, advocacy and community mobilization were central to the success of the programme (Sanghvi *et al*. 2016).

Similarly, Haselow *et al.* review the evolution and impact of the enhanced homestead food production (EHFP) programme, which aims to increase year‐round availability and intake of diverse nutrient‐rich foods while promoting optimal feeding and nutrition practices in poor households. Programme evaluation in Bangladesh, Cambodia, Indonesia, Nepal and the Philippines indicates that EHFP had a positive impact on poor households' year round food production, food consumption – particularly among women and children 6–59 months of age – and food security. Results from randomized and non‐randomized programme evaluations in Bangladesh and Nepal have shown significant improvements in a range of practices known to impact positively child growth, with reductions in child stunting ranging from 10.5% to 18.0% (Haselow *et al*. 2016).

It is clear from this set of papers that despite the challenges, there are good programme examples in South Asia that may be replicated and scaled up to accelerate improvements in IYCF. These must, however, be adapted to new settings other than their originating context, and more evidence must be built around these adaptations to strengthen the regional evidence base.

## Improving women's nutrition outcomes

There is broad agreement that direct maternal nutrition interventions are needed to improve women's nutrition, birth outcomes and children's linear growth in infancy and early childhood. However, there are few programme examples – beyond those related to improving antenatal care services during pregnancy – that are explicitly focused on improving women's nutrition before, during or after pregnancy. For South Asia this is a crucial area of investment as it looks ahead.

In addition, over the last decade a number of studies have documented the role of non‐nutrition determinants on women's nutrition and the nutrition of their offspring. In her paper, Vir reviews the evidence on the impact of different dimensions of women's agency such as age at marriage and conception, formal education, decision making power relative to men and control over resources, on the nutrition of women and children. Vir concludes that combining direct nutrition interventions with measures that empower women is essential. A range of programme platforms in sectors such as health, education, agriculture, employment, microfinance and social protection can be used strategically to improve women's access to food, health and care before and during pregnancy and lactation as well as to strengthen women's knowledge, skills and agency to feed and care for their children (Vir 2016).

## The challenge of bringing together nutrition and sanitation

It is becoming increasingly clear that all post‐natal stunting cannot be completely reversed by improving children's diets if children live in highly unhygienic environments. Growing evidence suggests that there is a link between children's linear growth and the sanitation practices in the households where children live. The ingestion of high quantities of faecal bacteria by young children through mouthing soiled fingers and household items leads to intestinal infections which affect children's nutritional status by diminishing appetite, reducing nutrient absorption, and increasing nutrient losses. Although the proportion of people using improved sanitation in South Asia increased by 18 percentage points between 1990 and 2011, the pace of this improvement has not kept up with population growth; as a result, the region accounts for almost two thirds of the global population practicing open defecation.

In this special issue, Cumming and Cairncross review how poor water, sanitation and hygiene (WASH) conditions can have a significant detrimental effect on child growth through sustained exposure to enteric pathogens and wider social and economic mechanisms. Mbuya and Humphrey argue that the unhygienic environments in which many infants and young children live cause an environmental enteric dysfunction (EED), whereby the children's guts have: (1) reduced absorptive capacity that results in nutrient maldigestion and malabsorption; and (2) reduced structural integrity (i.e. a ‘leaky gut’) that results in chronic immune activation and the diversion of nutrients to fight infection rather than growth. These two overlapping pathways result in poor linear growth and stunting (Cumming & Cairncross 2016; Mbuya & Humphrey 2016). In light of the above, there is renewed interest in how WASH interventions might be targeted or modified to support efforts in the Nutrition sector.

The evidence of how WASH interventions might support nutrition interventions to improve linear growth and reduce stunting is still ‘under construction’. Large WASH intervention studies are currently underway both in rural and urban settings to add to this evidence base. Cumming and Cairncross argue that the main challenge for nutrition‐sensitive WASH strategies is to ensure that populations with a high burden of stunting are targeted before or when growth faltering occurs and with appropriate WASH interventions alongside nutrition‐specific interventions (Cumming & Cairncross 2016). A recent Cochrane review reporting that the effect of WASH interventions on child stunting was greatest in children aged 0–23 months validates this focus (Dangour *et al*. 2013). Mbuya and Humphrey suggest that a package of baby‐WASH interventions (sanitation and water improvement, handwashing with soap, ensuring food hygiene and a clean infant feeding and play environment) to interrupt feco‐oral transmission in the first two years of a child's life may make important contributions to global efforts to reduce stunting where poor sanitation is a major challenge (Mbuya & Humphrey 2016).

## Cost and benefits of reducing stunting

Two major considerations for the delivery of interventions to improve child feeding, women's nutrition and household sanitation are the costs and benefits of investing in these outcomes. In this special issue, the review by Shekar *et al*. on the costs and benefits of reducing stunting indicates that the global public investment required to scale up critical nutrition interventions globally is estimated at US$ 10.3 billion above and beyond existing investments. Such investment would reduce the number of stunted children by about 30 million and save at least 1.1 million lives. Shekar *et al.* argue that rigorous estimations of the costs and benefits of nutrition investments to children and societies are an important next step in South Asian countries. Such cost‐benefit analyses will help identify the reduction of stunting as a national development and investment priority, drive political commitment and action, and ramp up the effective allocation of resources to reduce child stunting (Shekar *et al*. 2016).

In an attempt to address this issue, Menon *et al.* estimate that in India US$ 4.5 billion/year are required to deliver the package of 10 nutrition‐specific interventions promoted by the Scaling Up Nutrition (SUN) movement, while US$ 5.9 billion/year are required to deliver the package of 14 nutrition specific interventions that are encompassed in India's nutrition policy frameworks, with an average cost of US$ 140 child/year. It is important to mention that maternity cash benefits (49%) and food 14 supplements (40%) contribute to almost 90% of the total cost. The authors conclude that there is an urgent need for further costing studies on the true unit costs of high‐impact nutrition interventions to be able to project accurate national and subnational budgets for nutrition in India (Menon *et al*. 2016).

## Country‐focused strategies to reduce stunting in South Asia: what will it take?

In developing this special issue, we requested planning, programme, and research specialists based in South Asia to share their reflections on child stunting in their countries (Higgins‐Steele *et al.* for Afghanistan; Ahmed *et al.* for Bangladesh; Dzed and Wangmo for Bhutan; Avula *et al.* for India; Das & Bhutta for Pakistan; and Devkota & Adhikari for Nepal). These perspective papers highlight similarities and challenges across South Asian countries. All papers emphasize the multisectoral nature of the interventions that are needed to reduce stunting and therefore, the need for high‐level leadership to enable the scale up of nutrition‐specific interventions as well as of investments to improve the underlying drivers of stunting in a nutrition‐sensitive manner, particularly food security, women's status, and hygiene and sanitation. The papers from Afghanistan, India and Pakistan also highlight the importance of sub‐national variability, and therefore the need for strategies that are specifically tailored to context so as to enable lagging regions to catch up. However, despite strong economic growth in much of the region, few of the country perspective papers reflect on the importance of advocating for a model of economic growth of fuels further public investments in social sectors, poverty reduction, increased equity and the role of non‐government stakeholders and private sector actors. Similarly, few of the country perspectives reflect on additional research needs to support a better understanding of what strategies might work better across the region. Our own perspective is that these areas – increased and equitable social sector investments, the role of non‐government actors and the importance of better research and knowledge – are crucial as there are context‐specific needs in terms of social investments, public–private partnerships, and research needs to support the scale up of nutrition‐specific interventions, to inform integration with nutrition‐sensitive interventions, and to strengthen political commitments to nutrition across the region.

## Stop stunting in South Asia: what is next?

As we mention in our introduction, this special issue of *Maternal and Child Nutrition* captures much of what was discussed in preparation for and during the Regional Conference: *Stop Stunting*: *Improving Child Feeding*, *Women*'*s Nutrition and Household Sanitation in South Asia*. As a summary to the rich discussions that took place during the conference, we requested Shawn Baker, Director for Nutrition at the Bill and Melinda Gates Foundation, to share his views about what works, what is missing, and what is next to stop stunting in South Asia. His views, in the form of 10 *take home messages* are a good conclusion to this overview paper.

### Message 1: Children from all regions of the world have similar potential for growth and development in early childhood

Child stunting is a powerful marker of failed development. In nations where stunting has declined many things have worked in favour of children. Conversely, wherever stunting remains high, development is failing children. Children's growth is a mirror of the state of a society and stunting is possibly the most sensitive indicator of overall societal equity and well‐being. Therefore, it makes perfect sense that child stunting be one of the lead nutrition indicators for the post‐2015 Sustainable Development Goals.

### Message 2: Stunting is an outrage that demands a response commensurate with the damage it is doing to children and nations

Stunting has declined in South Asia but still compromises the future of 38% of underfives – almost 65 million children – and the future of the region as a whole. Good physical growth and brain development are every child's birth right. Stunted children do not have a voice and their plight is so ubiquitous that it is viewed as the ‘normal’ state of affairs. However, evidence from within and outside the region proves that large scale declines in stunting – for millions of children at a time – can be achieved.

### Message 3: We need to create ‘a new normal’ for the drivers of child stunting in South Asia

This new state of affairs needs to comprise a new normal for child feeding that includes age‐appropriate foods for infants and young children, and ensures quality, quantity and safety; a new normal for women's lives that includes good nutrition, healthy height, healthy weight, no anaemia and the right to make decisions affecting their lives; and finally, a new normal for household hygiene and sanitation practices that includes access to safe water and sanitation, washing with soap at critical times, and the end of open defecation.

### Message 4. South Asian countries can afford to act and cannot afford the cost of inaction

Evidence shows that economic growth alone will not improve stunting without commensurate investments in other accompanying interventions. We need to move from expecting that economic growth will ‘trickle down’ to making strategic investments on evidence‐based large scale programmes that place their emphasis of the most vulnerable children and populations. South Asian countries need to seize the opportunity of economic growth to invest in the future of children. It will cost, but it is an investment that ‘locks in the potential’, with benefits that far exceed the cost.

### Message 5. The one‐thousand days from conception to age two years are a key window in which interventions to prevent stunting should focus

A growing number of national nutrition programmes are responding to the challenge of child stunting by focusing on the *golden* one‐thousand days and ensuring that children under two years of age and their mothers meet their nutrient needs. Nutrient density and diet diversity for children and women are of the essence. Evidence shows that they can be improved at scale using a mix of interventions that includes locally‐available foods, fortified foods, and supplementary foods where food insecurity is a problem.

### Message 6: Act now and for the future. Multiple drivers need multiple actions

It is essential that we deliver known solutions at scale to address the underlying causes of stunting: child feeding, women's nutrition and household sanitation. However, it will be crucial that we partner with kindred spirits to address the more distal and inter‐generational drivers of child stunting in South Asia: adolescent marriage and pregnancy, women's illiteracy and poor decision making power, and household poverty and social exclusion. It will be essential to define the roles and responsibilities of each sector in reducing child stunting and, importantly, to co‐locate the interventions of all sectors.

### Message 7: We need to start with focus and scale in mind

The response to child stunting in South Asia needs to be commensurate with the scale of the problem. Multiple platforms can be used to deliver the interventions that will stop stunting: antenatal care visits, institutional deliveries, adolescent‐focused programmes, mother‐and‐child services, home visits and community‐based programmes, social protection schemes and women's micro‐credit programmes are a few examples. Scaling up improved hygiene and sanitation practices and ending open defecation will be essential to ensure that improved nutrient intakes result into improved growth outcomes.

### Message 8. Know your epidemic, know your response

The drivers of child stunting change in nature and intensity from country to country and within countries. Therefore it is important to have an accurate understanding of what is driving poor nutrition in children and women (availability, access, use, knowledge, choice, markets, poverty, exclusion…) and poor hygiene and sanitation practices in the household (infrastructure, beliefs, norms, tradition, gender, awareness…) to tailor a response that brings about a significant decline in child stunting. In all instances it will be crucial that national strategies prioritize the most vulnerable children: the youngest, the poorest and the socially excluded.

### Message 9: Engage strategically with and monitor carefully the private sector

The private sector – commercial and non‐for‐profit – is an increasingly large player in the sectors that affect child stunting: food, health, water, sanitation, education, communication and employment. National and sub‐national governments have the opportunity to optimize the potential added value of and mitigate the potential harm from the private sector by establishing quality standards, enforcing adequate regulation and legislation measures, and ensuring competition. The expertise of the non‐food private sector in supply chain and logistics, mobile technology and mass media communication, demand creation, and capacity building could potentially be used to support interventions but this will need further exploration.

### Message 10: Serious problems require serious measurement

There is no doubt that we need a *data revolution* if we are serious about reducing stunting in South Asia. National and sub‐national governments need to collect more frequent and more disaggregated data to measure progress towards the World Health Assembly nutrition goals, including child stunting, as well as the coverage of effective interventions in different age, gender, geographic and socio‐economic groups. Measuring the performance of national systems in delivering essential interventions to prevent stunting and tracking investments and expenditures against costed plans ensure public accountability and indicate good governance.

## Conclusion

Nutrition is key to children's survival, growth and development. Well‐nourished children are healthier and cleverer than their undernourished peers, they grow and develop to their full potential and they perform better in school and as adults. Despite significant progress over the last two decades, an estimated 38% of children under the age of five in South Asia are stunted because of persistent nutrition deprivation. Besides being a moral imperative, investing to prevent stunting in South Asia is a development imperative. It is our hope that the wealth of research and programmatic evidence included in this special issue of *Maternal and Child Nutrition* will inform the post‐2015 drive to stop stunting in South Asia.

## Conflicts of interest

The authors declare that they have no conflicts of interest. The opinions expressed on this paper are those of the authors and do not necessarily represent an official position by UNICEF or IFPRI.

## Contributions

Both authors contributed to manuscript writing and have read and approved the final submission.
